# Psychometric properties of the Brazilian version of Pfeffer's Functional Activities Questionnaire

**DOI:** 10.3389/fnagi.2014.00255

**Published:** 2014-09-25

**Authors:** Luciana de Oliveira Assis, Jonas J. de Paula, Marcella G. Assis, Edgar N. de Moraes, Leandro F. Malloy-Diniz

**Affiliations:** ^1^Post Graduation Program in Neuroscience, Federal University of Minas GeraisBelo Horizonte, Brazil; ^2^Faculty of Humanities, Social Sciences and Health, FUMEC UniversityBelo Horizonte, Brazil; ^3^Department of Psychology, Faculty of Medical Sciences of Minas GeraisBelo Horizonte, MG, Brazil; ^4^Faculty of Medicine, National Institute of Science and Technology – Molecular Medicine, Federal University of Minas GeraisBelo Horizonte, Brazil; ^5^Department of Occupational Therapy, Faculty of Physical Education, Physiotherapy and Occupational Therapy, Federal University of Minas GeraisBelo Horizonte, Brazil; ^6^Department of Clinics, Faculty of Medicine, Federal University of Minas GeraisBelo Horizonte, Brazil; ^7^Laboratory of Neuropsychological Investigations (LIN), Universidade Federal de Minas GeraisBelo Horizonte, Brazil; ^8^Department of Mental Health, Faculty of Medicine, Federal University of Minas GeraisBelo Horizonte, Brazil

**Keywords:** functional assessment, older adult, instrumental activities of daily living, psychometric properties, neuropsychology, validity, reliability

## Abstract

Pfeffer's Functional Activities Questionnaire (FAQ) is one of the most commonly employed tools in studies on pathological cognitive aging. Despite the different versions of the questionnaire translated for use in clinical practice, few studies have analyzed the psychometric properties of the Brazilian version of the FAQ (P-FAQ). Thus, the aim of the present study was to analyze the P-FAQ with regard to internal consistency, factorial structure and associations with demographic factors (age, sex, and schooling), depressive symptoms, cognitive measures and other measures of functionality. One hundred sixty-one older adults were divided into four groups (91 with dementia, 46 with mild cognitive impairment, 11 with psychiatric disorders and 13 healthy controls). All participants were evaluated by cognitive, behavioral and functional tests and scales. Their caregivers answered the P-FAQ. The questionnaire showed high internal consistency (α = 0.91). Factor analysis revealed a two-factor structure, which, accounted for 66% of the total variance. The P-FAQ was not correlated with demographic factors, was weakly correlated with depressive symptoms (ϱ = 0.271, *p* < 0.01, *R*^2^ = 7%) and strongly correlated with cognitive measures (Matttis Dementia Rating Scale total score: ϱ = −0.574, *p* < 0.01, *R*^2^ = 33%) as well as complex instrumental activities of daily living (ϱ = −0.845, *p* < 0.01, *R*^2^ = 71%). Cognitive performance and depression status were independent predictors of P-FAQ scores in regression models. The present findings indicate that the P-FAQ has satisfactory reliability, internal consistency, construct validity and ecological validity. Therefore, this questionnaire can be used in clinical practice and research involving the Brazilian population of older adults.

## Introduction

The proportion of older adults in the general population has increased in recent years due mainly to the demographic explosion in past decades as well as improvements in living conditions and quality of life (Lin et al., 2012). With the increase in life expectancy, disabling diseases associated with the aging process have become more prevalent.

Functional status is one of the most important aspects of geriatric evaluations and extremely relevant to diagnostic procedures, as atypical cognitive and behavioral manifestations often stem from normal aging. Moreover, neuropsychiatric disorders, such as dementia (Lopes and Bottino, 2002), depression and psychosis (Hoffmann et al., 2010) are characterized by persistent cognitive and functional dysfunction, resulting in limitations that worsen with the progression of the disease. The formal diagnosis of dementia requires the adequate characterization of functional impairment, which is non-existent or less impacting in conditions such as mild cognitive impairment (Petersen et al., 2001; Yassuda et al., 2010; Brown et al., 2011; de Paula and Malloy-Diniz, 2013). Thus, evidence of functional impairment constitutes an important indicator of pathological aging (Freitas and Miranda, 2011).

The use of questionnaires that evaluate basic and instrumental activities of daily living is a common method for evaluating the functional status of older adults. Basic activities include self-care, toileting, eating, dressing, bathing, hygiene, functional locomotion and sphincter control, whereas instrumental activities are those related to enjoying an independent, active life, such as household chores, managing finances, taking medication, running errands as well as using transportation and the telephone.

Despite the importance of scales for the evaluation of functionality, few functional status measures employed in Brazil have been submitted to formal adaptation and validation procedures for use on older adults (Vasconcelos et al., 2007). Pfeffer's Functional Assessment Questionnaire (FAQ) is one of the most widely used measures of functional status in research and is often employed in epidemiological studies on dementia (Nitrini et al., 2004; Laks et al., 2005, 2010; Aprahamian et al., 2011). The interest of researchers in different centers on the questionnaire has grown in recent years, especially after its inclusion in the assessment protocol of the Alzheimer's Disease Neuroimaging Initiative (2014). This questionnaire is particularly useful due to its potential in discriminating individuals with and without cognitive impairment (Devanand et al., 2008; Steenland et al., 2008). Moreover, the FAQ exhibits greater sensitivity (0.85) in comparison to the Lawton Scale (0.57) when used to distinguish individuals with and without dementia (Pfeffer et al., 1982).

The FAQ was formally adapted to the Brazilian context in a recent study (Sanchez et al., 2011), although other versions with subtle differences have been used in clinical and research contexts. While the translated version, denominated the Pfeffer's Functional Activities Questionnaire (P-FAQ), has similar characteristics to Pfeffer's original questionnaire, a number of items have been completely changed, with the possible alteration of the original structure. However, no previous studies have evaluated the psychometric properties of the P-FAQ on a heterogeneous sample of older Brazilian adults.

Thus, the aim of the present study was to analyze the P-FAQ with regard to internal consistency, factorial structure and associations with demographic factors (age, sex, and schooling), depressive symptoms, cognitive measures and other measures of functionality.

## Materials and methods

### Subjects

The participants were aged 60 years or older and recruited from the Jenny de Andrade Faria Institute of Healthcare for Older Adults and Women, which is a secondary/tertiary public health center in the city of Belo Horizonte, Brazil. This institute receives older adults referred from primary healthcare units in metropolitan Belo Horizonte as well as other municipalities in the state of Minas Gerais. The participants were sent for neuropsychological exams as part of routine evaluations or follow up and were subsequently invited to participate in the present study. A total of 161 older adults (96 women and 65 men; mean age: 75.51 ± 7.22 years; mean schooling: 4.43 ± 4.08 years) were included in the study.

This project integrates a comprehensive study which aims to evaluate the psychometric properties of a neuropsychological protocol designed to assess older adults with low formal education (de Paula et al., 2013a). The project was approved by the Research Ethics Committee of the Federal University of Minas Gerais (COEP-334/06). All participants and/or legal guardians signed a statement of informed consent. Individuals with severe sensory or motor impairment or without caregivers to provide information were excluded from the study.

### Participants

The cognitive evaluation involved the Mini Mental State Examination (Folstein et al., 1975), the Brazilian version of the Mattis Dementia Rating Scale (MDRS) and its five subscales (Porto et al., 2003), the Clock Drawing Test (Shulman, 2000) and one of the Brazilian versions of the Frontal Assessment Battery (de Paula et al., 2013b). These measures were selected for representing different aspects of cognition (general and specific), involving language, memory, visuospatial skills, attention and executive functions, as recommended in previous studies (Salmon and Bondi, 2009; Weintraub et al., 2009). All aforementioned measures have been cross-culturally adapted and validated for use on the Brazilian population (de Paula et al., 2010, 2013a).

The participants were also evaluated with regard to psychiatric symptoms, involving the administration of the Brazilian version of the 15-item Geriatric Depression Scale (GDS-15) (Almeida and Almeida, 1999) and an interview with open-ended answers on functional status for the determination of functional complaints based on caregivers' reports focused on lost skills. The Clinical Dementia Rating (CDR) (Morris, 1993) was used to determine the stage of dementia. Only individuals with a CDR score of 1 or less were included in the study. The diagnosis was performed by consensus among a geriatrician, psychiatrist and neuropsychologist. The clinical evaluation of the geriatrician also involved an interview with the participant and caregiver to investigate symptoms, disease progression, functional loss, family history and possible confounders. Clinical and neuroimaging exams were performed when necessary.

Following the descriptive evaluations, the participants were allocated to different groups based on the clinical condition: dementia [*n* = 91; 71 with mild to moderate dementia (probable Alzheimer's disease); five with frontotemporal dementia; four with vascular dementia; and three with mixed dementia); mild cognitive impairment (46 with amnesic mild cognitive impairment); psychiatric disorder (*n* = 11; nine with a diagnosis of depression and two with late-onset psychosis); and healthy controls (13 individuals with no disorders that could affect cognition or behavior).

### P-FAQ

The P-FAQ is a version of the FAQ that is frequently employed in Brazil in both clinical practice and research (Ministério da Saúde, 2007; Jacinto, 2008; Moraes, 2008; Brito, 2010; Hoffmann et al., 2010; Damin, 2011; Lino, 2011; Jacinto et al., 2012). This questionnaire allows the evaluation of the degree of independence on the performance of ten instrumental activities of daily living: managing one's own finances; shopping; heating water and shutting off the stove; making meals; keeping track of current events, watching news reports and discussing them; maintaining oneself orientated when walking outside the neighborhood; remembering commitments; managing one's own medications; and being at home alone (Moraes, 2008). The last three items on the P-FAQ differ from the original version of the FAQ: remembering appointments and taking care of one's own medication; playing cards or performing other hobbies; and dealing with business or documents. The scoring was the same, with the total score ranging from 0 to 30 points (worst performance). Caregivers also answered the General Activities of Daily Living (GADL) Scale, which is divided into self-care activities, domestic activities and complex activities, as described elsewhere (de Paula et al., 2014).

### Statistical procedures

The sample size was calculated using the G^*^Power program, version 3.1.7. As the Kolmogorov-Smirnov test demonstrated that most data exhibited non-normal distribution, a sample of 161 individuals was considered adequate to detect large (98%), moderate (93%) and small (73%) effect sizes in the comparisons of non-parametric groups. Descriptive statistics were performed for the demographic characteristics of the participants as well as the scores on the Mini-Mental State Examination, Frontal Assessment Battery, MRDRS, Clock Drawing Test, GADL scale and Geriatric Depression Scale. Differences among the four groups (dementia, mild cognitive impairment, psychiatric disorders and control) were analyzed using the Kruskall-Wallis test, followed by the Mann-Whitney tests with the Bonferroni correction for group-by-group analyses. The chi-square test was used to determine differences among categorical variables.

The validity of the P-FAQ was evaluated using exploratory factor analysis of the ten items. Principal axis factoring and varimax rotation were selected for the procedure. Eigenvalues greater than 1 and scree plot analysis, the latter of which was performed by two independent observers (JJP and LFMD), were employed for the selection of the factors. Based on the sample size, factor loadings equal to or greater than 0.45 were considered significant (Hair et al., 2009).

Internal consistency of the P-FAQ was investigated using Cronbach's alpha coefficient. Spearman's non-parametric correlation coefficients were calculated to determine associations between the questionnaire and socio-demographic (age and schooling), cognitive (Mattis scale, Mini Mental Health Examination, Clock Drawing Test and Frontal Assessment Battery), neuropsychiatric (Geriatric Depression Scale) and functional (three components of the GADL scale) measures. Coefficients of determination were calculated for the analysis of shared variance among these variables. A forced-entry multiple regression model was used for the evaluation of the main predictors of the P-FAQ score. To minimize the collinearity of the model, only the total Mattis score, age, schooling, sex and depressive symptoms were used as predictors. All statistical procedures were conducted using the SPSS 17.0 (SPSS Inc., 2008).

## Results

Table 1 displays the description of the socio-demographic, functional, psychiatric and cognitive characteristics of the participants. The different groups were similar with regard to age, schooling and activities of daily living related to self-care. Significant differences were found in the proportion of men to women (χ^2^ = 8.23; *p* = 0.041). The psychiatric disorder group had a larger proportion of women than the other three groups. Significant differences were also found for the other variables analyzed.

**Table 1:** **Description of groups according to socio-demographic, functional, cognitive and psychiatric variables**.

	**Control (1)**	**Mild cognitive impairment (2)**	**Dementia (3)**	**Psychiatric disorder (4)**	**KW**	***Post-hoc***
	**Median (25th–75th percentile)**	**Median (25th–75th percentile)**	**Median (25th–75th percentile)**	**Median (25th–75th percentile)**		
Age	79	74	76	77	2.00	–
Schooling	4 (4–11)	3 (2–4)	4 (1–4)	4 (0–5)	6.67	–
Female gender (*n*)	7	25	53	11	–	–
P-FAQ	0 (0–2)	4 (1–8)	14 (9–19)	12 (2–15)	51.63^**^	1 < 2, 1 < 3, 1 < 4, 2 < 3
GADL—self-care	8 (8–8)	8 (8–8)	8 (8–8)	8 (8–8)	2.38	–
GADL—domestic activities	8 (8–8)	8 (7–8)	6 (4–8)	6 (5–8)	36.53^**^	1 > 3, 1 > 4, 2 > 3, 2 > 4
GADL—complex activities	8 (7–8)	7 (6–8)	4 (2–7)	7 (2–8)	44.25^**^	1 > 3, 1 > 4, 2 > 3
Geriatric depression scale	2 (0–3)	3 (1–4)	4 (2–6)	8 (5–11)	20.09^**^	1 < 3, 1 < 4, 2 < 3, 2 < 4, 3 < 4
Mini-mental state examination	27 (23–28)	25 (20–27)	20 (17–23)	22 (19–26)	28.16^**^	1 > 3, 2 > 3
frontal assessment battery	15 (12–17)	12 (10–13)	8 (6–11)	8 (6–13)	40.28^**^	1 > 2, 1 > 3, 1 > 4, 2 > 3
Clock drawing test	5 (3–5)	2 (1–4)	2 (0–3)	3 (2–4)	19.05^**^	1 > 2, 1 > 3
MDRS attention	36 (35–36)	35 (33–36)	34 (32–35)	35 (34–36)	19.27^**^	1 > 3, 2 > 3
MDRS I/P	34 (31–37)	29 (25–31)	23 (21–28)	26 (22–29)	34.20^**^	1 > 2, 1 > 3, 1 > 4,2 > 3
MDRS construction	6 (6–6)	6 (4–6)	5 (2–6)	6 (3–6)	9.99^*^	1 > 3
MDRS conceptualization	33 (32–37)	32 (27–35)	24 (21–31)	28 (22–37)	26.50^**^	1 > 3, 2 > 3
MDRS memory	23 (22–24)	18 (16–21)	13 (10–17)	18 (13–20)	39.20^**^	1 > 2, 1 > 3, 1 > 4, 2 > 3
MDRS total	131	118	102	115	53.82^**^	1 > 2, 1 > 3, 1 > 4, 2 > 3

*Significant difference at p < 0.01; KW, Kruskall-Wallis test; P-FAQ, Pfeffer's Functional Activities Questionnaire; GADL, General Activities of Daily Living Scale; MDRS, Mattis Dementia Rating Scale; I/P, Initiative/Perseveration. 1 – interpreted as: 0.02–0.12 (low), 0.13–0.25 (medium), 0.26 or higher (high)*.

* p < 0.05;

** *p < 0.01*.

The results of the Kaiser-Meyer-Olkin Measure of Sampling Adequacy (KMO = 0.889) and Bartlett's Sphericity Test (χ^2^ = 929.48; *p* < 0.001) suggest that the sample was appropriate for factor analysis of the P-FAQ. Following the extraction of the factors and orthogonal rotation of the data, a two-factor structure was considered the most suitable for the data (Table 2). The first factor explained 55% of the variance (eigenvalue: 5.50) and the second factor explained approximately 11% of the overall variance (eigenvalue: 1.07). The latent structure therefore suggests bi-factor distribution.

**Table 2:** **Rotated factor structure (varimax) of P-FAQ**.

**Components**	**Factor loadings**	
	**Factor 1**	**Factor 2**
Is he/she capable of walking outside the neighborhood and finding the way back home?	**0.704**	0.295
Is he/she capable of buying clothes, food and other things by himself/herself?	**0.690**	0.405
Is he/she capable of making a meal?	**0.689**	0.230
Is he/she capable of heating water for coffee and turning off the stove?	**0.647**	0.313
Does he/she manage his/her money?	**0.579**	0.361
Is he/she capable of managing his/her medications?	**0.566**	**0.448**
Is he/she capable of remembering appointments, family events and holidays?	**0.562**	**0.532**
Can he/she be left alone at home safely?	**0.512**	0.119
Is he/she capable of paying attention, understanding and discussing a radio or television program, newspaper or magazine?	0.238	**0.934**
Is he/she capable of keeping track of current events and occurrences in the community or neighborhood?	0.366	**0.771**

*P-FAQ: Pfeffer's Functional Activities Questionnaire*.

*Significant factor loadings according to sample size*.

The P-FAQ exhibited high internal consistency (α = 0.91). The correlation analyses suggest that the P-FAQ was not correlated with age or schooling in the present sample. Significant correlations were found between the questionnaire and the three components of the GADL: a small effect size was found for the self-care component and large effect sizes were found for the domestic and complex components, with more than 70% shared variance with the latter component. A significant association, albeit with a small effect size, was found between the P-FAQ and depressive symptoms. Correlations between the questionnaire and cognitive measures ranged from weak to strong. The strongest correlations were found with general cognition (MDRS total score) and executive functions (Mattis Initiative/Perseveration). Weak correlations were found with measures of visuospatial skills (Clock Drawing Test and MDRS Construction). The other correlations between the P-FAQ and cognitive measures exhibited a moderate effect size (Table 3).

**Table 3:** **Spearman's correlation coefficients and shared variance (*R*^2^) between P-FAQ and socio-demographic, cognitive, functional and psychiatric variables**.

**Variable**	**ϱ**	***R*^2^(%)**
Age	0.117	<1
Schooling	−0.109	<1
GADL self-care	−0.232^**^	5
GADL domestic activities	−0.687^**^	47
GADL complex activities	−0.845^**^	71
Geriatric depression scale	0.271^**^	7
Mini mental state examination	−0.420^**^	18
Frontal assessment battery	−0.440^**^	19
Clock drawing test	−0.260^**^	7
MDRS attention	−0.361^**^	13
MDRS I/P	−0.537^**^	29
MDRS construction	−0.239^**^	6
MDRS conceptualization	−0.388^**^	15
MDRS memory	−0.457^**^	21
MDRS total	−0.574^**^	33

*Significant to p < 0.01; P-FAQ, Pfeffer's Functional Activities Questionnaire; GADL, General Activities of Daily Living Scale; MDRS, Mattis Dementia Rating Scale; I/P, Initiative/Perseveration*.

** *p < 0.01*.

**Figure 1:**
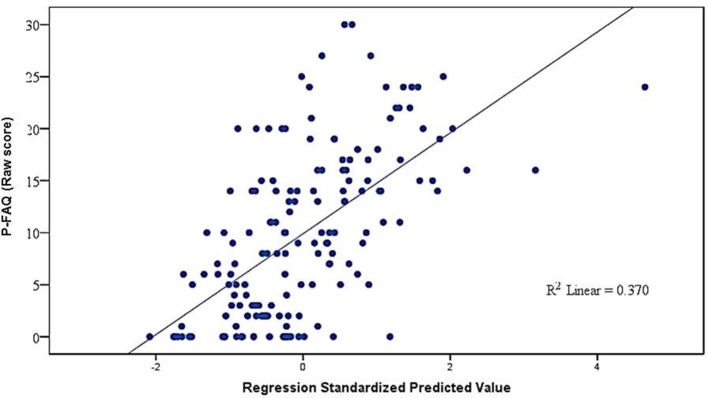
**Multiple linear regression of P-FAQ score, socio-demographic characteristics, cognition and depressive symptoms**. P-FAQ: Pfeffer's Functional Activities Questionnaire.

The multiple regression model designed for the determination of predictors of functional performance was significant [*F*_(5, 155)_ = 17.68; *p* < 0.001; adjusted *R*^2^ = 34%]. The significant predictors were the MDRS total score (β = −0.234; *SE* = 0.03; *p* < 0.001) and Geriatric Depression Scale (β = 0.426; *SE* = 0.15; *p* = 0.007). Marginally significant predictors were schooling (β = 0.271; *SE* = 0.14; *p* = 0.054) and the female sex (β = 1.818; *SE* = 1.073; *p* = 0.092), but not age (β = 0.072; *SE* = 0.07; *p* = 0.330). Figure 1 displays the relationship between standardized predictors and performance on the P-FAQ.

## Discussion

The present findings demonstrate the psychometric adequacy of the P-FAQ in terms of reliability and validity. Moreover, the questionnaire demonstrated satisfactory internal consistency (Cronbach's α = 0.91). Although this version has three items that differ from the original FAQ, the items that comprise the P-FAQ are homogeneous, maintaining the internal consistency of the questionnaire, and possibly interchangeable. These results are similar to data described by Sanchez et al. (2011), who report α = 0.95 in the administration of the scale to a sample of older Brazilian adults.

The factorial structure of the P-FAQ exhibited two components. The first incorporated complex instrumental activities of daily living, with the largest loading factor found for “*capable of walking outside the neighborhood and finding the way back home,”* followed by *“capable of buying clothes, food and other things by himself/herself.”* The second factor addresses activities strongly related to planning and prospective memory, which are considered complex activities, but possibly with different cognitive and procedural demands. The findings demonstrate the construct validity of the questionnaire, with two factors associated with complex activities. Moreover, the correlations were stronger for complex instrumental activities involving greater cognitive involvement in comparison to basic routine activities of a domestic nature. These results are in agreement with data reported in the original study by Pfeffer et al. (1982), who considers the items on the FAQ to be more complex than those on previous scales, such as that proposed by Lawton et al. (Lawton and Brody, 1969).

A heterogeneous correlation pattern was found between the P-FAQ and the cognitive, functional and psychiatric tests selected for the present study. The strongest correlations were found for a global cognitive variable (MDRS total score) and a variable related to executive functions (Mattis Initiative/Perseveration) and moderate correlations were found for more general executive functions (Frontal Assessment Battery) and a cognitive screening test (Mini-Mental State Examination). These findings are in agreement with data described in a previous study, in which executive functions and functional performance were strongly correlated in a similar population (de Paula and Malloy-Diniz, 2013). Greenaway et al. (2012) also found the MDRS to be a predictor of functional decline in older adults. The present findings are in agreement with data described in a review of the literature conducted by Royall et al. (2007), in which measures of executive functions and general cognition were more strongly associated with performance on activities of daily living. It should be stressed that the Mattis Initiative/Perseverance subscale involves verbal fluency tasks that depend on both executive functions and processing speed (de Paula et al., 2013c), the latter of which has been associated with functional performance in studies with heterogeneous populations (Brown et al., 2013).

Moderate correlations were found between the P-FAQ and tasks related to memory (Mattis Memory), language/semantic memory (Mattis Conceptualization) and attention/work memory (Mattis Attention), suggesting that such aspects of cognition play a secondary role in the performance of complex activities of daily living. The weakest correlations found between cognitive and functional measures were related to visuospatial skills (Mattis Construction and Clock Drawing Test). However, previous studies have found significant associations between functional performance and visuospatial skills (Davies et al., 2011; Farley et al., 2011). This divergence reflects the need for components directed at the evaluation of activities strongly related to the processing of spatial information. The P-FAQ has only one item addressing this aspect (*“Is he/she capable of walking outside the neighborhood and finding the way back home?”*), which, however, is strongly influenced by other cognitive aspects, such as non-declarative memory (habits and procedural memory).

Depressive symptoms constituted another significant predictor of functional performance in the present study. These symptoms were estimated using a scale that has been validated for the Brazilian population (Almeida and Almeida, 1999). Although the association was weak, depressive symptoms were independently associated with cognitive and socio-demographic aspects. Such symptoms are important determinants of functional decline in older adults (Hoffmann et al., 2010; de Paula, 2012; de Paula et al., 2013c), but can be understood as either a cause or consequence of functional decline, which is an aspect that should be analyzed further in future studies.

Significant differences were found among the different groups evaluated using the P-FAQ, the largest of which were between the healthy controls and patients with dementia. Significant differences were also found among the healthy controls, patients with mild cognitive impairment and those with psychiatric disorders as well as between patients with mild cognitive impairment and those with dementia. Analyzing healthy older adults, those with mild cognitive impairment and those with dementia, Jacinto (2008), also found that the P-FAQ demonstrated sufficient efficacy in the diagnosis of cognitive decline. The capacity of the FAQ to distinguish health older adults from those with dementia (Pfeffer et al., 1982) gives the questionnaire clinical importance (Alzheimer's Disease Neuroimaging Initiative, 2014). The P-FAQ also has this characteristic. Further studies should be conducted to evaluate the possible additive of effect between this version of the FAQ and cognitive measures for the differential diagnosis of pathological aging, as performed with another functional scale used as a parameter in the present investigation.

The present study has limitations that should be addressed. The participants were grouped in general categories (dementia, mild cognitive impairment and psychiatric disorders) without considering subdivisions, such as Alzheimer's disease and frontotemporal dementia in the group of patients with dementia, since the sample size is relatively small. As functional impairment may differ among these patients, the present findings cannot be directly transposed to these specific groups. The comparison of an ecological parameter for the evaluation of the FAQ, which is the gold standard for functional assessments, would allow a more accurate analysis of the ecological validity of the questionnaire (Chaytor and Schmitter-Edgecombe, 2003).

## Funding

This work was supported by the following grants: APQ-01972/12-10, APQ-02755-10, APQ-04706-10, CBB-APQ-00075-09 from FAPEMIG, and 573646/2008-2 from CNPq. The funders had no role in study design, data collection, analysis, decision to publish, or preparation of the manuscript.

### Conflict of interest statement

The authors declare that the research was conducted in the absence of any commercial or financial relationships that could be construed as a potential conflict of interest.
